# Autonomic neurotransmission in cardiovascular regulation and pathophysiology

**DOI:** 10.3389/fnins.2025.1739330

**Published:** 2026-01-26

**Authors:** Fahimeh Varzideh, Stanislovas S. Jankauskas, Pasquale Mone, Urna Kansakar, Gaetano Santulli

**Affiliations:** 1 Department of Molecular, Cellular and Biomedical Sciences, School of Medicine, City University of New York, Manhattan, NY, United States; 2 Casa di Cura Clinica Montevergine, Avellino, Italy; 3 International Translational Research and Medical Education (ITME) Consortium, Joint Academic Research Unit, Naples, Italy; 4 Division of Cardiology, Department of Medicine, Albert Einstein College of Medicine, Wilf Family Cardiovascular Research Institute, Einstein Institute for Neuroimmunology and Inflammation (INI), New York, NY, United States; 5 Department of Molecular Pharmacology, Albert Einstein College of Medicine, Einstein-Mount Sinai Diabetes Research Center (ES-DRC), Einstein Institute for Aging Research, Fleischer Institute for Diabetes and Metabolism (FIDAM), New York, NY, United States

**Keywords:** acetylcholine, autonomic nervous system, brain, cardiovascular disease, heart rate variability, neuro-cardiology, neurotransmission, sympathetic nervous system

## Abstract

The autonomic nervous system (ANS) is a central regulator of cardiovascular function, coordinating involuntary control of heart rate, vascular tone, and blood pressure through its sympathetic (SNS) and parasympathetic (PNS) subdivisions. The SNS mediates the “fight or flight” response via catecholamines, increasing heart rate, contractility, and vasoconstriction, whereas the PNS promotes restorative processes through acetylcholine, decreasing heart rate and enhancing vasodilation. Nitric oxide further modulates vascular tone and autonomic balance, serving as a key neuromodulator. Assessment of cardiovascular autonomic function relies on heart rate variability, baroreflex sensitivity, and other physiological tests, which provide insight into the dynamic interplay between sympathetic and parasympathetic activity. Dysregulation of the ANS contributes to cardiovascular pathologies, including cardiovascular autonomic neuropathy, hypertension, and heart failure, where sympathetic overactivity and impaired parasympathetic modulation exacerbate disease progression. Pharmacologic interventions, such as β-blockers and ivabradine, alongside non-pharmacologic approaches, including structured exercise and respiratory training, aim to restore autonomic balance and improve clinical outcomes. Understanding the exact mechanisms of autonomic neurotransmission is essential for identifying novel therapeutic targets and optimizing cardiovascular care. Future research integrating molecular, genetic, and systems-level analyses will further elucidate autonomic regulation, guiding personalized interventions to mitigate cardiovascular morbidity and mortality.

## Introduction

The autonomic nervous system (ANS) is a key component of the peripheral nervous system that regulates involuntary physiological functions essential for maintaining homeostasis. This complex neural network controls heart rate, blood pressure, digestion, and respiration without conscious input. In cardiovascular physiology, the ANS is fundamental to the modulation of cardiac activity and vascular tone through its two major subdivisions: the sympathetic and parasympathetic nervous systems (SNS, PNS, Table 1). Herein we examine the mechanisms of autonomic neurotransmission in cardiovascular regulation, emphasizing their roles in health and disease.

**TABLE 1:** Main differences between sympathetic and parasympathetic nervous system and cardiovascular implications.

Parameter	Sympathetic nervous system (SNS)	Parasympathetic nervous system (PNS)	Clinical implications
Primary neurotransmitter	Norepinephrine (NE); epinephrine (from adrenal medulla)	Acetylcholine (ACh)	SNS overactivity can be managed with β-blockers, α-blockers; PNS modulation with vagal maneuvers or atropine if excessive vagal tone causes bradycardia
Receptors	Adrenergic (α1, α2, β1, β2, β3)	Muscarinic (mainly M2 in the heart)	β-blockers inhibit β1-mediated cardiac effects; muscarinic antagonists block excessive vagal activity
Origin of fibers	Thoracolumbar (T1–L2) spinal cord	Craniosacral (medulla oblongata and sacral spinal cord)	Knowledge of fiber origin guides regional interventions (e.g., stellate ganglion block in arrhythmias)
Ganglia location	Near spinal cord (paravertebral/prevertebral)	Near or within target organs (intramural)	Ganglion-targeted therapies (SNS: sympathectomy; PNS: vagal nerve stimulation)
Heart rate (chronotropy)	Increases via β1 at SA node	Decreases via M2 at SA node	Tachyarrhythmias can be mitigated by β-blockers; bradyarrhythmias may require atropine or pacing
Conduction velocity (dromotropy)	Increases (AV node facilitation)	Decreases (AV node inhibition)	AV nodal conduction disorders can be influenced pharmacologically (β-blockers vs. anticholinergics)
Contractility (inotropy)	Increases (β1-mediated Ca^2^? influx)	Slight decrease (mostly atria)	Positive inotropes target SNS pathways; excessive SNS activation can worsen HF
Vascular effects	Vasoconstriction via α1; vasodilation in skeletal muscle via β2	Minimal direct effect; some vasodilation via NO	Hypertension treated with α- or β-blockers; PNS modulation may aid endothelial function
Blood pressure	Elevates (↑ CO and TPR)	Lowers (↓ HR and slight vasodilation)	SNS blockade lowers BP; enhancing vagal tone can assist BP regulation
Coronary circulation	Moderate vasoconstriction (α) but metabolic vasodilation predominates	Vasodilation via endothelial NO release	Coronary ischemia may benefit from β-blockers; NO-mediated PNS pathways protect endothelium
Heart rate variability (HRV)	Low HRV indicates sympathetic dominance	High HRV reflects vagal tone and cardiovascular health	HRV-guided therapy: exercise, β-blockers, or vagal nerve stimulation to restore autonomic balance
Response to stress/exercise	“Fight or flight” – ↑ CO, BP, oxygen delivery	“Rest and digest” – restores homeostasis post-stress	Stress-related cardiovascular events may be prevented by controlling SNS overactivation
Clinical correlations	Hypertension, HF, arrhythmias, myocardial ischemia	Sinus bradycardia, vasovagal syncope, high vagal tone in athletes	β-blockers for HF and arrhythmias; pacing for bradycardia; lifestyle interventions to enhance HRV
Therapeutic targets	β-blockers, α-blockers, sympatholytics	Muscarinic antagonists (atropine), vagal nerve stimulation	Pharmacologic and non-pharmacologic interventions aim to restore autonomic balance in CV disease

## Structure of the ANS

### SNS

The SNS governs acute cardiovascular responses, particularly under conditions of stress or physical exertion, but it also plays a fundamental role in normal cardiovascular physiology at rest. Through the tonic and phasic release of catecholamines, the SNS continuously contributes to the regulation of heart rate, myocardial contractility, vascular tone, and blood pressure, ensuring moment-to-moment cardiovascular stability. Even in the absence of external stressors, basal sympathetic activity is essential for maintaining resting arterial pressure, supporting venous return, and preserving adequate organ perfusion. During situations requiring increased metabolic demand—such as exercise, emotional stress, or orthostatic challenge—sympathetic outflow is rapidly augmented. This results in accelerated heart rate, enhanced myocardial contractility, and peripheral vasoconstriction, collectively increasing cardiac output and arterial pressure. These adaptive responses enable rapid redistribution of blood flow to vital organs and working muscles, forming the physiological basis of the classic “fight or flight” response (Dergacheva et al., 2013; Elia and Fossati, 2023). Importantly, the SNS operates in continuous dynamic balance with parasympathetic influences, allowing fine-tuned cardiovascular control across a wide range of physiological states. Thus, sympathetic activation should not be viewed solely as a stress-evoked mechanism, but rather as an integral component of both basal cardiovascular homeostasis and adaptive cardiovascular responses.

Catecholamines act on adrenergic receptors in the heart and vasculature. Activation of β1-adrenergic receptors increases heart rate (positive chronotropic effect) and contractility (positive inotropic effect), while stimulation of α1-adrenergic receptors in vascular smooth muscle induces vasoconstriction, elevating systemic vascular resistance (Lataro et al., 2013; Li et al., 2009). Sympathetic modulation of cardiovascular activity is often inferred non-invasively through heart rate variability (HRV) analysis, which captures beat-to-beat fluctuations in sinus rhythm arising from the dynamic interplay between autonomic inputs (Kasahara et al., 2021). However, HRV primarily reflects cardiac autonomic modulation and is heavily influenced by parasympathetic (vagal) activity, particularly in the time-domain indices and the high-frequency spectral component. Even frequency-domain measures traditionally interpreted as sympathetic markers, such as low-frequency (LF) power or the ratio LF/high-frequency (LF/HF), do not provide a direct or specific quantification of sympathetic nerve firing and are now recognized as integrative, context-dependent indices rather than true surrogates of sympathetic tone. In contrast, microneurography represents the gold-standard technique for direct assessment of sympathetic neural outflow, allowing real-time recording of muscle sympathetic nerve activity (MSNA) in peripheral nerves. This method provides a direct, quantitative measure of sympathetic discharge frequency and burst amplitude, and has been instrumental in defining the pathophysiological role of sympathetic overactivity in cardiovascular disease (Mano et al., 2006). The absence of microneurographic data therefore limits the ability to draw definitive conclusions regarding sympathetic nerve activity *per se*. Accordingly, HRV should be interpreted as an indirect and predominantly vagally weighted marker of autonomic balance rather than a precise measure of sympathetic activity. While HRV remains valuable for non-invasive, large-scale, and longitudinal studies, its limitations must be acknowledged, and conclusions regarding sympathetic modulation should be framed cautiously unless supported by direct techniques such as microneurography or complementary measures of sympathetic function.

### PNS

The PNS predominates during rest and recovery, promoting cardiovascular relaxation and energy conservation. Its primary neurotransmitter, acetylcholine, released from vagal efferents, acts on muscarinic receptors in the heart, particularly M2 receptors in the sinoatrial node. Activation of these receptors hyperpolarizes pacemaker cells, resulting in bradycardia (negative chronotropic effect) and vasodilation in select vascular territories (Makiguchi et al., 2009; Song et al., 2022). This vagal influence restores cardiovascular equilibrium following sympathetic activation and enhances HRV, a marker of parasympathetic dominance. The PNS plays a crucial role in cardiovascular recovery and health, with dysfunction linked to a higher risk of arrhythmias, hypertension, and heart failure (HF) (de Andrade et al., 2023; Olshansky et al., 2008).

### Integration of sympathetic and parasympathetic activity

Effective cardiovascular regulation depends on the balance between sympathetic and parasympathetic influences. During exercise or acute stress, sympathetic activation elevates heart rate and blood pressure to meet metabolic demands (Sharma et al., 2011), while parasympathetic dominance during rest lowers cardiac activity and supports digestion. This dynamic equilibrium, often described as autonomic balance, is reflected in HRV measurements (Silvani et al., 2016). Persistent sympathetic overactivity disrupts this balance, contributing to hypertension, HF, and other cardiovascular pathologies (Griffioen et al., 2007).

## Neurotransmitters in cardiovascular regulation

### Catecholamines

Catecholamines, primarily norepinephrine and epinephrine, are central mediators of autonomic cardiovascular control. Released from sympathetic nerve terminals and the adrenal medulla, these hormones enable rapid cardiovascular adjustments to stress. Norepinephrine increases heart rate, contractility, and vascular tone through β1- and α1-adrenergic receptor activation, thereby augmenting cardiac output and blood pressure. Epinephrine exerts similar effects and also modulates metabolism by increasing plasma glucose during stress (Gasser and Daws, 2017). Dysregulated catecholamine signaling contributes to hypertension, HF, and arrhythmogenesis (Santulli and Iaccarino, 2016; Scalco et al., 2024).

### Acetylcholine

Acetylcholine, the primary parasympathetic neurotransmitter, modulates heart rate and vascular tone through muscarinic receptor activation. Stimulation of M2 receptors in the sinoatrial node reduces heart rate, while activation of endothelial receptors promotes nitric oxide–mediated vasodilation (MacDonald et al., 2020; Grzêda et al., 2023). This mechanism counterbalances catecholaminergic excitation and stabilizes cardiovascular function. Reduced parasympathetic tone, as observed in chronic stress or metabolic disease, predisposes individuals to tachycardia and cardiovascular instability (Ahriţculesei et al., 2025; Shanks et al., 2023).

### Nitric oxide

Nitric oxide (NO), produced from L-arginine by endothelial nitric oxide synthase, is a key vasodilator and modulator of autonomic activity (Gambardella et al., 2020). It modulates sympathetic nervous system (SNS) activity at multiple, integrated levels, acting as a physiological counter-regulatory signal that restrains excessive sympathetic vasoconstriction and cardiovascular excitation. At the central nervous system level, NO functions as a neuromodulator within key autonomic control regions, including the nucleus tractus solitarius, rostral ventrolateral medulla, and hypothalamus. In these areas, NO derived from neuronal and endothelial nitric oxide synthase dampens sympathetic premotor neuron activity, thereby reducing sympathetic outflow to the heart and peripheral vasculature. This central inhibitory influence contributes to baroreflex sensitivity and limits sympathetic activation during both basal conditions and stress responses. At the peripheral neurovascular interface, NO attenuates sympathetic neurotransmission by inhibiting norepinephrine release from postganglionic sympathetic nerve terminals. Through cGMP-dependent mechanisms, NO reduces calcium influx in presynaptic neurons, blunting vesicular catecholamine release and diminishing neurogenic vasoconstriction. This effect is particularly relevant during exercise and acute stress, when sympathetic drive is elevated but excessive vasoconstriction must be avoided to preserve tissue perfusion (Tsuru et al., 2002; Ufnal and Sikora, 2011). Within the vascular wall, NO directly counteracts SNS-mediated vasoconstriction by inducing relaxation of vascular smooth muscle cells via soluble guanylate cyclase activation and cGMP generation (Gordon and Sved, 2002; Gambardella et al., 2020). This postjunctional action reduces vascular responsiveness to α-adrenergic stimulation, effectively buffering the pressor effects of catecholamines. As a result, NO allows appropriate increases in blood flow without disproportionate rises in blood pressure. Additionally, NO influences autonomic reflex control, enhancing arterial baroreflex function and promoting sympathoinhibition in response to increases in blood pressure. Impairment of endothelial NO bioavailability therefore leads not only to endothelial dysfunction but also to heightened sympathetic tone, creating a feed-forward cycle that promotes hypertension, vascular remodeling, and atherosclerosis (Soda et al., 2023; Młynarska et al., 2025). Thus, NO acts as a critical integrator of vascular and autonomic regulation, ensuring that sympathetic activation during physiological challenges is appropriately scaled and spatially controlled, thereby preserving cardiovascular homeostasis and protection.

## Assessment of cardiovascular autonomic function

### HRV

Heart rate variability quantifies fluctuations in the intervals between heartbeats and serves as a sensitive marker of autonomic control. High HRV indicates robust parasympathetic modulation, while low HRV reflects sympathetic dominance and is associated with stress, metabolic disease, and adverse cardiovascular outcomes (Kapa et al., 2016; Arakaki et al., 2023; Ziemssen and Siepmann, 2019). Reduced HRV is common in diabetes and hypertension, highlighting the importance of autonomic assessment in these conditions (Hadaya and Ardell, 2020; Haque and Dutta, 2025; Lefrandt et al., 2010; Touyz et al., 2018). Despite this strong physiological and prognostic relevance, HRV remains underutilized in routine cardiology practice. Several factors help explain why many cardiologists tend to overlook HRV. First, HRV indices are indirect markers of autonomic modulation rather than direct measures of sympathetic nerve activity, and their physiological interpretation, particularly of frequency-domain parameters such as the LF/HF ratio, has been debated. This has contributed to skepticism regarding their clinical specificity and utility. Second, HRV is highly context-dependent and influenced by respiration, posture, circadian rhythm, physical activity, medications, and intrinsic HR, necessitating standardized acquisition and careful interpretation that are not always practical in routine clinical workflows. In addition, although reduced HRV is consistently associated with increased cardiovascular and all-cause mortality, HRV has not been fully incorporated into guideline-based decision-making, partly because interventional studies demonstrating that HRV-guided management improves clinical outcomes are limited. Consequently, HRV is often regarded as a research or epidemiological tool rather than a clinically actionable biomarker. Technical challenges, including heterogeneity in analytical methods and limited harmonization across devices and software platforms, further restrict its widespread adoption. Nevertheless, the frequent observation of reduced HRV in diabetes, hypertension, and other cardiometabolic disorders highlights its value as an integrative marker of autonomic and cardiovascular health. When interpreted within a rigorous methodological framework and in conjunction with conventional clinical measures, HRV can provide complementary information that enhances risk stratification and deepens understanding of autonomic dysfunction in cardiovascular disease.

### Baroreflex sensitivity

Baroreflex sensitivity maintains blood pressure homeostasis by adjusting heart rate and vascular resistance in response to arterial pressure changes. Baroreceptors in the carotid sinus and aortic arch detect vessel stretch, initiating autonomic reflexes that restore blood pressure equilibrium (Suarez-Roca et al., 2021). Hypertension and diabetes frequently impair this mechanism, resulting in blunted reflexes and increased cardiovascular risk (Lefrandt et al., 2010; Scridon, 2022; Woodard et al., 2002).

### Methodological approaches

Autonomic function is assessed through both time-domain (e.g., SDNN, RMSSD) and frequency-domain analyses of HRV. Frequency-domain parameters distinguish sympathetic (low-frequency) and parasympathetic (high-frequency) influences (Liu et al., 2022; Tahsili-Fahadan and Geocadin, 2017). Additional tests, such as tilt-table and pharmacologic evaluations, provide insights into autonomic adaptability under controlled challenges (Jason et al., 2024; Yugar et al., 2023).

## Autonomic dysregulation in cardiovascular disease

### Cardiovascular autonomic neuropathy

Cardiovascular autonomic neuropathy (CAN) is a serious microvascular complication of diabetes mellitus, characterized by progressive impairment of autonomic control over heart rate, blood pressure, and vascular tone (Duque et al., 2021). The pathophysiology of CAN is multifactorial, with chronic hyperglycemia playing a central role by inducing oxidative stress, endothelial dysfunction, and direct neuronal injury, ultimately leading to degeneration of both sympathetic and parasympathetic fibers (Jankauskas et al., 2021). Clinically, CAN manifests as resting tachycardia, reduced HRV, exercise intolerance, and orthostatic hypotension (Verrotti et al., 2009). These manifestations often appear insidiously, with early-stage CAN remaining asymptomatic, which underscores the challenge of timely diagnosis.

Despite its subclinical onset, CAN is a strong predictor of cardiovascular morbidity and mortality in diabetic populations, as impaired autonomic modulation contributes to arrhythmias, silent myocardial ischemia, and sudden cardiac death (Liu et al., 2020; Rajan and Gokhale, 2002). The prevalence of CAN increases with diabetes duration and poor glycemic control, emphasizing the importance of early screening. Routine evaluation using non-invasive measures, such as HRV analysis, baroreflex sensitivity testing, and orthostatic blood pressure assessment, is recommended to detect autonomic dysfunction before overt cardiovascular complications develop (Kadoi, 2010). Early identification and intervention may mitigate disease progression and improve long-term outcomes in individuals with diabetes.

### Hypertension

Hypertension is a prevalent cardiovascular disorder closely linked to autonomic dysregulation, characterized by heightened sympathetic activity and reduced parasympathetic tone (Rajan and Gokhale, 2002; Yeh et al., 2023). Persistent sympathetic overactivation increases peripheral vascular resistance through α1-adrenergic–mediated vasoconstriction and promotes structural changes in the vasculature, including smooth muscle hypertrophy and arterial stiffening, which contribute to sustained elevations in blood pressure (Duprez, 2008). Concomitantly, parasympathetic withdrawal diminishes the ability of the heart to counterbalance sympathetic effects, further exacerbating cardiovascular strain. Beyond sympathetic overactivation, parasympathetic withdrawal represents a critical and distinct contributor to blood pressure elevation and cardiovascular risk. Several mechanisms underlie parasympathetic withdrawal in hypertension. Impaired arterial baroreflex sensitivity is a central factor, as chronic elevations in blood pressure reset baroreceptor function toward higher operating pressures, reducing vagal efferent signaling to the sinoatrial node. This blunted baroreflex-mediated parasympathetic output limits the heart’s ability to buffer transient increases in blood pressure and heart rate, thereby favoring sympathetic dominance. Neurohumoral activation further contributes to vagal suppression. Elevated circulating levels of angiotensin II and aldosterone, common in hypertension, exert inhibitory effects on central parasympathetic nuclei while simultaneously facilitating sympathetic outflow. Angiotensin II also interferes with acetylcholine release at cardiac vagal terminals, directly reducing parasympathetic influence on HR regulation. Metabolic and inflammatory factors play additional roles. Insulin resistance, oxidative stress, and low-grade inflammation (frequent features of hypertension) impair central autonomic integration and reduce vagal tone through altered neurotransmission and neuronal excitability. Endothelial dysfunction and reduced nitric oxide bioavailability may further weaken parasympathetic modulation by disrupting afferent signaling from cardiovascular mechanoreceptors. Collectively, parasympathetic withdrawal diminishes the heart’s capacity to counterbalance sympathetic vasoconstrictive and chronotropic effects, leading to reduced heart rate variability, impaired cardiovascular adaptability, and increased myocardial oxygen demand. This loss of vagal restraint amplifies cardiovascular strain and accelerates target-organ damage, underscoring the importance of restoring autonomic balance as a therapeutic objective in hypertension.

Impaired baroreflex sensitivity, commonly observed in hypertensive individuals, compounds this imbalance by reducing the capacity of the ANS to buffer fluctuations in blood pressure, creating a deleterious feedback loop that perpetuates hypertension and increases the risk of target organ damage, including left ventricular hypertrophy, renal dysfunction, and cerebrovascular events (Narkiewicz and Grassi, 2008). Resistant hypertension, defined as blood pressure that remains elevated despite the use of multiple antihypertensive agents, frequently arises in the setting of severe autonomic dysfunction (Carey et al., 2018; Schiffrin and Fisher, 2024). Management of such cases often requires multifaceted strategies combining pharmacologic interventions—including β-blockers, α-blockers, and renin-angiotensin system inhibitors—with non-pharmacologic measures such as lifestyle modification, exercise, and stress reduction (Dinh et al., 2011; Gogan et al., 2025; Mazzeo et al., 2013). Addressing autonomic imbalance is therefore critical to achieving optimal blood pressure control and reducing cardiovascular morbidity and mortality.

### HF

High-frequency is characterized by a profound imbalance in autonomic regulation, with heightened sympathetic drive and diminished parasympathetic (vagal) tone contributing to hemodynamic instability and progressive cardiac dysfunction (Hansen et al., 2018). Chronic sympathetic overactivation increases heart rate and systemic vascular resistance, which elevates myocardial oxygen demand and workload, thereby exacerbating ventricular remodeling and functional decline. Reduced vagal activity limits the heart’s capacity to counteract these effects, further destabilizing cardiac performance (Olshansky et al., 2008; Zhang and Anderson, 2014).

Impaired baroreflex sensitivity, a hallmark of autonomic dysfunction in HF, diminishes the body’s ability to regulate blood pressure and heart rate in response to physiological stress, and is strongly associated with adverse prognosis (Lefrandt et al., 2010; Tang et al., 2014). Moreover, the presence of CAN in HF patients accelerates functional deterioration and is linked to increased morbidity and mortality, emphasizing the additive detrimental effect of autonomic neuropathy on an already compromised cardiovascular system (Gözüküçük et al., 2024; Olivieri et al., 2024).

Therapeutic strategies aimed at restoring autonomic balance have shown promise in improving outcomes. Interventions that enhance parasympathetic activity—such as structured aerobic exercise, optimized blood pressure management, and, in selected cases, device-based neuromodulation—can increase HRV, reduce sympathetic overactivity, and improve overall cardiovascular performance (Kay et al., 2022; Giquel et al., 2012). Addressing autonomic dysfunction is therefore a critical component of comprehensive HF management, with the potential to slow disease progression and reduce mortality.

### Arrhythmias

Autonomic imbalance plays a pivotal role in the initiation and maintenance of cardiac arrhythmias. Enhanced sympathetic activity increases automaticity, shortens the refractory period, and promotes after depolarizations, which collectively heighten susceptibility to atrial and ventricular arrhythmias. Conversely, diminished parasympathetic (vagal) tone reduces protective modulation of cardiac excitability, facilitating arrhythmogenic triggers. Imbalances in autonomic input can exacerbate conditions such as atrial fibrillation, ventricular tachycardia, and sudden cardiac death, particularly in patients with underlying structural heart disease or HF (Talishinsky et al., 2025). HRV and baroreflex sensitivity are commonly used as surrogate markers to assess autonomic contribution to arrhythmic risk, with reduced HRV and impaired baroreflex function strongly correlating with increased incidence of adverse cardiac events (La Rovere et al., 2001). Interventions targeting autonomic modulation, including β-blockers, catheter-based sympathetic denervation, and vagal nerve stimulation, have demonstrated efficacy in reducing arrhythmic burden by restoring autonomic balance and stabilizing cardiac electrophysiology (Chung et al., 2023; van Weperen et al., 2021). Understanding the interplay between sympathetic and parasympathetic influences is therefore essential for preventing and managing arrhythmias in high-risk cardiovascular populations.

## Clinical implications and future directions

### Therapeutic strategies

Targeting the ANS has emerged as a central component in the management of cardiovascular diseases. Pharmacologic interventions primarily aim to modulate sympathetic overactivity or enhance parasympathetic tone. Beta-adrenergic receptor blockers (β-blockers) are widely employed to attenuate excessive sympathetic stimulation, effectively reducing heart rate, myocardial oxygen demand, and systemic vascular resistance, which translates into improved clinical outcomes and survival in patients with hypertension, heart failure, and certain arrhythmias (Latic et al., 2022). Additionally, agents such as ivabradine selectively lower heart rate by enhancing parasympathetic influence on the sinoatrial node without adversely affecting myocardial contractility (Szczeklik et al., 2025), providing benefit in patients with heart failure or high resting heart rates (Pinto et al., 2025). Pharmacologic interventions primarily aim to restore autonomic balance by attenuating sympathetic overactivity or enhancing parasympathetic tone. Beta-adrenergic receptor blockers (β-blockers) remain the cornerstone therapy for conditions associated with heightened sympathetic drive. By antagonizing β1-adrenergic receptors in the heart, they reduce HR, decrease myocardial contractility, and lower systemic vascular resistance, collectively diminishing myocardial oxygen demand and mitigating adverse remodeling (Latic et al., 2022). These effects translate into improved clinical outcomes and survival in patients with hypertension, heart failure, and certain arrhythmias, while also modulating autonomic function by reducing sympathetic outflow and partially restoring vagal activity. In parallel, agents such as ivabradine selectively inhibit the If current in the sinoatrial node, lowering heart rate without negatively impacting myocardial contractility. Evidence suggests that ivabradine not only reduces heart rate but may enhance parasympathetic influence, improve heart rate variability, and promote more balanced autonomic control, particularly in patients with heart failure or elevated resting heart rates (Szczeklik et al., 2025). By combining these pharmacologic strategies, clinicians can target both arms of autonomic regulation—dampening harmful sympathetic overdrive while supporting protective parasympathetic activity—ultimately optimizing cardiovascular outcomes.

Non-pharmacological strategies complement pharmacologic therapy by promoting autonomic balance and cardiovascular resilience. Structured aerobic exercise, yoga, and respiratory training enhance parasympathetic activity, reduce sympathetic dominance, and increase HRV, thereby improving both functional capacity and overall cardiovascular outcomes (Yuen et al., 2018).

Combining pharmacologic and lifestyle interventions provides a synergistic approach to restoring autonomic equilibrium, mitigating disease progression, and improving prognosis. Tailoring these strategies to individual patient profiles, guided by autonomic function assessments such as HRV and baroreflex sensitivity, may optimize therapeutic efficacy and reduce cardiovascular morbidity and mortality.

### Future directions

The field of neuro-cardiology is rapidly evolving, with growing recognition of the central role of autonomic neurotransmission in cardiovascular health and disease. Future research is likely to focus on elucidating the molecular, genetic, and cellular mechanisms underlying autonomic dysregulation, enabling identification of novel therapeutic targets to restore sympathetic-parasympathetic balance. Advances in precision medicine may allow for individualized interventions based on patient-specific autonomic profiles, integrating data from HRV, baroreflex sensitivity, and other biomarkers to tailor pharmacologic and non-pharmacologic therapies.

Innovative pharmacologic approaches are being explored to selectively modulate autonomic pathways, including agents that enhance parasympathetic tone or selectively inhibit maladaptive sympathetic overactivity without compromising essential cardiovascular function (Ogino et al., 2004). Device-based neuromodulation, such as vagal nerve stimulation, baroreflex activation, and targeted sympathetic denervation, offers promising adjunctive strategies for patients with heart failure, resistant hypertension, or arrhythmias.

Additionally, behavioral, environmental, and lifestyle factors—including stress management, exercise, and diet—are increasingly recognized as modulators of autonomic function, highlighting the need for integrative approaches that combine molecular, clinical, and lifestyle interventions. Longitudinal studies integrating multi-omics data, neuroimaging, and autonomic assessment will provide deeper insights into the dynamic interactions between the nervous system and cardiovascular physiology. Ultimately, these efforts aim to refine therapeutic strategies, improve patient outcomes, and prevent the progression of cardiovascular diseases associated with autonomic dysfunction (Esler et al., 2002).

## Conclusion

Autonomic neurotransmission is central to cardiovascular homeostasis. The coordinated actions of the sympathetic and parasympathetic systems regulate heart rate, vascular tone, and blood pressure. Dysregulation, as seen in conditions such as CAN, hypertension, and heart failure, contributes to adverse outcomes and heightened mortality. Understanding these mechanisms has guided therapeutic development, emphasizing pharmacologic modulation of autonomic tone and supportive lifestyle interventions. Future work integrating mechanistic, clinical, and personalized approaches promises to refine treatments and improve cardiovascular resilience through restoration of autonomic balance.
